# Characteristics of bilirubin photochemical changes under green light-emitting diodes in humans compared with animal species

**DOI:** 10.1038/s41598-021-85632-5

**Published:** 2021-03-18

**Authors:** Kohichiroh Nii, Hitoshi Okada, Susumu Itoh, Takashi Kusaka

**Affiliations:** Department of Pediatrics, Kagawa University, 1750-1 Ikenobe, Miki-cho, Kitagun, Kagawa 761-0793 Japan

**Keywords:** Biochemistry, Chemical biology, Biophysical chemistry, Experimental models of disease, Paediatric research, Lasers, LEDs and light sources

## Abstract

Phototherapy using light-emitting diodes (LEDs) centered on the green spectrum, which has a high cyclobilirubin production rate, was as effective as that centered on the blue spectrum for neonatal hyperbilirubinemia. There are no reports of species differences in bilirubin photochemical changes in this spectrum, and the characteristics of bilirubin photochemical changes in humans must be elucidated to proceed with the development of new light sources that include these spectra. This report describes the characteristic photochemical kinetics of bilirubin under green-spectrum LEDs in human, rat, rabbit, dog, pig, sheep, bovine and chicken serum albumin and rhesus monkey serum. These albumin-bilirubin complex solutions were irradiated by green LEDs, and the time-course changes in bilirubin photoisomers were measured by high-performance liquid chromatography. The cyclobilirubin production rates in humans, pigs, and monkeys were significantly higher than those in other species. The rate constant of *(EZ)*-cyclobilirubin production from *(EZ)*-bilirubin ‘k’ was significantly higher in humans and monkeys than in other species. In conclusion, bilirubin photochemical kinetics under green spectrum LEDs in humans were characterized by a high cyclobilirubin production rate at a low substrate concentration. The bilirubin photochemical kinetics in monkeys were similar to those in humans.

## Introduction

Phototherapy has been used in routine clinical practice to treat neonatal hyperbilirubinemia for more than 50 years^1^. Although the photochemical reactions of bilirubin have been elucidated, the development of a highly effective and safe light source is important. Blue-spectrum light sources including the maximal absorption wavelength of *4Z,15Z*-bilirubin (*(ZZ)*-bilirubin), 450–460 nm, have been used for phototherapy in many cases. When *(ZZ)*-bilirubin absorbs light, photoisomerization generates photoisomers that are less lipophilic than *(ZZ)*-bilirubin, and the photooxidation reaction produces the colorless low-molecular-weight products methylvinylmaleimide, hematinic acid and propentdyopents. Photoisomerization includes a configurational isomerization, namely, a Z(cis)-E(trans) isomerization reaction, by which *(ZZ)*-bilirubin forms *(ZE)*-bilirubin or *(EZ)*-bilirubin, and a structural photoisomerization that generates *(EZ)*-cyclobilirubin from *(EZ)*-bilirubin. These photoisomers are excreted into bile without undergoing glucuronidation. Bilirubin elimination depends on the production rate and the excretion of these products, and photooxidation is a slow process and a minor mechanism for neonatal phototherapy^2^.

In humans, the production and excretion of cyclobilirubin (lumirubin) are important mechanisms in bilirubin elimination^3^, and the optimal wavelength for cyclobilirubin production is 490–520 nm^4–7^. Although the effective spectrum of phototherapy is 430–490 nm, according to the American Academy of Pediatrics guidelines^8^, phototherapy using turquoise fluorescent tubes and green light-emitting diodes (LEDs) centered on 490^9^, 497^10^ or 500 nm^11^ was reported to be as effective as that using fluorescent tubes and LEDs centered on the blue spectrum. As many of the reported adverse reactions to phototherapy involved light sources centered on the blue spectrum, it may be possible to reduce adverse reactions by using a light source centered on the green spectrum. To develop a new light source centered on the green spectrum of 490–520 nm, it is important to evaluate the efficacy of phototherapy in humans for bilirubin reduction due to photochemical reactions, including cyclobilirubin production. It was previously reported that bilirubin photochemical reactions in humans differ from those in some animal species^12^, including Gunn rats, which are commonly used as an experimental animal model for hyperbilirubinemia^13^. During phototherapy, the main form excreted into bile in Gunn rats was *(ZE)*-/*(EZ)*-bilirubin ^14,15^, while that in human newborn infants was *(EZ)*-/*(EE)*-cyclobilirubin^3^. Therefore, Gunn rats cannot be used as an experimental animal model of human neonatal hyperbilirubinemia under phototherapy. However, differences among animal species have not been investigated using a green light source. To develop new light sources for phototherapy using animal models for human neonatal hyperbilirubinemia, it is necessary to clarify the differences in bilirubin photochemical reactions, including cyclobilirubin production, among animal species under both green and blue light sources. As the photochemical reactions of bilirubin are due to differences in albumin, we investigated these photochemical reactions, including cyclobilirubin production, in humans and several animal species.

## Results

Bilirubin photoisomers were produced 1 min after light irradiation in human serum albumin (SA), monkey serum, rat SA, rabbit SA, dog SA, pig SA, sheep SA, bovine SA, chicken SA, and dimethyl sulfoxide (DMSO) solution. Cyclobilirubin increased with irradiation time. *(ZE)*-Bilirubin and *(EZ)*-bilirubin rapidly increased at 1 min after irradiation. The *(ZE)*-bilirubin/*(ZZ)*-bilirubin ratio and *(EZ)*-bilirubin/*(ZZ)*-bilirubin ratio were constant from 1 to 3 min. Therefore, *(ZE)*-bilirubin and *(EZ)*-bilirubin were in a photoequilibrium state with *(ZZ)*-bilirubin.

**Figure 1:**
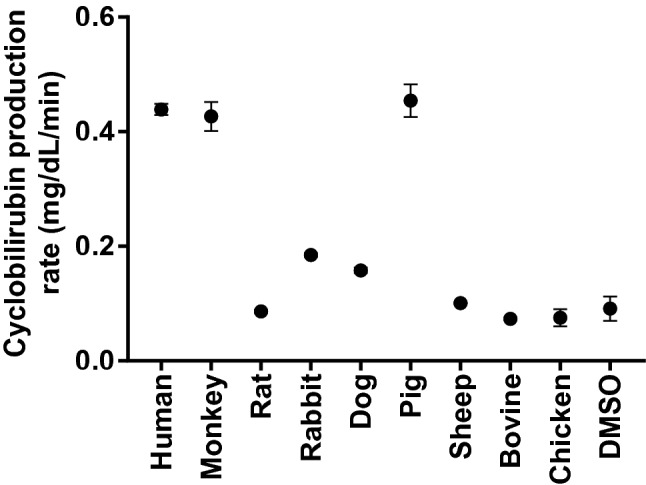
Cyclobilirubin production rate. The cyclobilirubin production rate at 3 min after irradiation. Cyclobilirubin is the sum of *(EZ)*-cyclobilirubin and *(EE)*-cyclobilirubin. Each closed circle and error bar represent the mean ± SD. DMSO, dimethyl sulfoxide.

The cyclobilirubin concentration and irradiation time were positively correlated (human SA: R^2^ = 0.96, p < 0.01, monkey serum: R^2^ = 0.99, p < 0.01, rat SA: R^2^ = 0.99, p < 0.01, rabbit SA: R^2^ = 0.99, p < 0.01, dog SA: R^2^ = 0.98, p < 0.01, pig SA: R^2^ = 0.98, p < 0.01, sheep SA: R^2^ = 0.99, p < 0.01, bovine SA: R^2^ = 0.99, p < 0.01, chicken SA: R^2^ = 0.92, p < 0.01, DMSO solution: R^2^ = 0.92, p < 0.01). The cyclobilirubin production rate [mean (1 standard deviation)] was 0.44 (0.01) mg/dL/min in human SA and ranged from 0.08–0.45 mg/dL/min in the other animal species (Fig. 1). The cyclobilirubin production rate in human SA was significantly higher than that in the SA of other species except for monkey serum (0.43 (0.03); p < 0.01) and pig SA (0.45 (0.03); p < 0.01).

**Figure 2:**
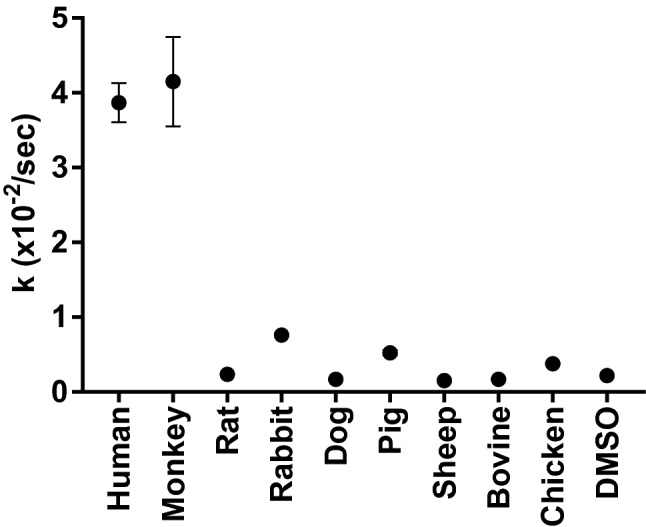
The rate constant of *(EZ)*-cyclobilirubin production from *(EZ)*-bilirubin (‘k’). The value of ‘k’ was calculated as the cyclobilirubin production rate divided by *(EZ)*-bilirubin at 3 min after irradiation. Each closed circle and error bar represent the mean ± SD. DMSO, dimethyl sulfoxide.

Here, ‘k’, the rate constant of *(EZ)*-cyclobilirubin production from *(EZ)*-bilirubin, was 3.9(0.3) × 10^–2^/s in human SA and ranged from 4.5 to 0.20 × 10^–2^ /sec in the SA of other species and in monkey serum (Fig. 2). The ‘k’ in human SA was significantly higher than that in the SA of other species, excluding monkey serum (4.2(0.6) × 10^–2^; p < 0.01).

**Figure 3:**
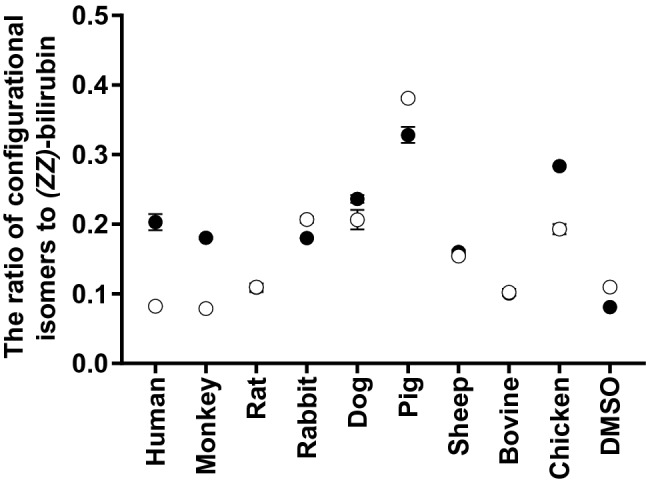
The ratio of configurational isomers to *(ZZ)*-bilirubin at photoequilibrium. The values of *(ZE)*-bilirubin and *(EZ)*-bilirubin were in a state of photoequilibrium with *(ZZ)*-bilirubin at 3 min. The closed circle represents the *(ZE)*-bilirubin/*(ZZ)*-bilirubin ratio, and the open circle represents the *(EZ)*-bilirubin/*(ZZ)*-bilirubin ratio. Each circle and error bar represent the mean ± SD. DMSO, dimethyl sulfoxide.

The *(ZE)*-bilirubin/*(ZZ)*-bilirubin ratio and *(EZ)*-bilirubin/*(ZZ)*-bilirubin ratio at photoequilibrium are shown in Fig. 3. The *(ZE)*-bilirubin/*(ZZ)*-bilirubin ratio was 0.20 (0.01) in human SA and ranged from 0.08 to 0.33 in monkey serum and in the SA of other species. The *(ZE)*-bilirubin/*(ZZ)*-bilirubin ratio in human SA was significantly different from that in SA of other species and in monkey serum (p < 0.01). The average ratios in monkey serum, rabbit SA, dog SA, and sheep SA were within the range of the average ratio in human SA by ± 0.5, but the average ratios in rat SA, pig SA, and chicken SA exceeded the ratio by +/− 1.0.

The *(EZ)*-bilirubin/*(ZZ)*-bilirubin ratio was 0.08 (0.01) in human SA and ranged from 0.08 to 0.38 in monkey serum and in the SA of other species. The *(EZ)*-bilirubin/*(ZZ)*-bilirubin ratio in human SA was significantly different from that in SA of other species, except in bovine SA and monkey serum (p < 0.01). The average ratios in monkey serum, rat SA, and bovine SA were within the range of the average ratio in human SA by ± 0.5, but the average ratios in rabbit SA, dog SA, pig SA, and chicken SA exceeded the ratio by + /− 1.0. In human SA, dog SA, chicken SA, and monkey serum, the ratio of *(ZE)*-bilirubin/*(ZZ)*-bilirubin was higher than that of *(EZ)*-bilirubin/*(ZZ)*-bilirubin. In rabbit SA and pig SA, the ratio of *(ZE)*-bilirubin/*(ZZ)*-bilirubin was lower than that of *(EZ)*-bilirubin/*(ZZ)*-bilirubin.

## Discussion

In this study, regarding the photochemical reaction when irradiating bilirubin bound to albumin with a light source centered on the green spectrum, the cyclobilirubin production rate differed among the animal species and was high in human SA, pig SA, and monkey serum. Among the animal SAs with a high cyclobilirubin production rate, some had a high ‘k’ and ratio of *(EZ)*-bilirubin/*(ZZ)*-bilirubin. In configurational isomerization, the ratio of configurational isomers to *(ZZ)-*bilirubin differed among animal species and was highest in pig SA.

For neonatal hyperbilirubinemia, the 430–490-nm spectrum has been used in phototherapy as an effective light source, and spectral irradiance was measured by radiometer across a wavelength of 425–475 or 400–480 nm^8^. In clinical practice, the effectiveness of turquoise and green light sources centered outside the valid wavelength range has also been reported^9–11^. As spectral irradiance has not been standardized even among currently available radiometers^16,17^, it is difficult to evaluate blue light sources, much less green light sources. Moreover, there are no reports on the characteristics of photochemical reactions in humans compared with animals using light sources centered on the green spectrum. Therefore, this study provides useful information for the future development of light sources centered on the green spectrum.

Structural isomerization is a reaction that produces *(EZ)*-cyclobilirubin from *(EZ)*-bilirubin (two-photon theory)^18,19^, although it has also been reported that *(EZ)*-cyclobilirubin is produced directly from *(ZZ)*-bilirubin (one-photon theory)^20^. *(ZZ)*-Bilirubin tetrapyrrole has the asymmetrical structure of a dipyrrole at the C_10_ methylene bridge, causing Z to E isomerization at C_4_ and C_15_ and cyclization of the endovinyl group at C_3_. On the other hand, configurational isomerization changes *(ZZ)*-bilirubin to *(ZE)*-bilirubin and *(EZ)*-bilirubin and changes *(EZ)*-cyclobilirubin to *(EE)*-cyclobilirubin. *(EZ)*-cyclobilirubin production depends on the light source wavelength and the number of absorbed photons^4–7,21^. When the reaction between *(ZE)*-/*(EZ)*-bilirubin and *(ZZ)*-bilirubin reaches photoequilibrium, the ratio of *(ZE)*-bilirubin and *(EZ)*-bilirubin to *(ZZ)*-bilirubin is a constant value corresponding to the light source wavelength, which is characteristic.

As a characteristic of bilirubin photochemical reactions in human SA solution, the *(EZ)*-cyclobilirubin production per absorbed photon from *(ZZ)*-bilirubin irradiated by a narrow band reached a maximum at 490–520 nm, and it decreased with shorter wavelengths to a minimum at 400–420 nm^4–7^. The relative ‘k’ under irradiation with a half-band width of 10 nm in human SA was mostly constant at 400–480 nm, increased at 480–500 nm, peaked at 500–520 nm, and then decreased at longer wavelengths^22^. On the other hand, the ratio of *(ZE)*-bilirubin to *(ZZ)*-bilirubin depended on the light source wavelength. *(ZE)*-bilirubin production irradiated by a half-band width of 10 nm was the highest at 400–420 nm and decreased with longer wavelengths, reaching a minimum at 500–520 nm in human SA-bilirubin solution^23^. Competitive inhibition of *(ZE)*-bilirubin and cyclobilirubin was suspected in excretion to bile^11^. After excretion to bile, *(ZE)*-bilirubin readily reacts to form *(ZZ)*-bilirubin, and some is reabsorbed through the gut^24^. In this study, we used an LED light source centered at 500 nm, which is used in clinical practice. The cyclobilirubin production rate was significantly higher in human SA, monkey serum, and pig SA than in the SA of the other species, whereas ‘k’ was significantly higher only in human SA and rhesus monkey serum. Under a blue-white fluorescent lamp, the cyclobilirubin production rate and ‘k’ in human SA were the highest among the reported species, including pig SA^12^. Under blue LEDs, the cyclobilirubin production rate was not significantly different between human SA and rhesus monkey serum^25^.

In this study, the *(ZE)*-bilirubin/*(ZZ)*-bilirubin ratio in human SA was lower than that under blue-white florescent lamp^12^ or blue LEDs^25^. Among animal SAs, the ratio was lower in rabbit SA and chicken SA, higher in dog SA, and similar in pig SA, bovine SA, and rat SA under a blue-white florescent lamp^12^. In rhesus monkey serum, the ratio was lower than that under blue LEDs^25^. The *(ZE)*-bilirubin/*(ZZ)*-bilirubin ratio in human SA was similar to the ratio in monkey serum, rabbit SA, dog SA, and sheep SA, higher than that in rat SA and bovine SA, and lower than that in pig SA and chicken SA. The difference in *(ZE)*-bilirubin/*(ZZ)*-bilirubin ratios among animals is considered to be due to the three-dimensional structure of bilirubin at the binding site of each animal albumin. In pigs and chickens, the effects of *(ZE)*-bilirubin on enterohepatic circulation were estimated to be higher than those in humans. In each animal species, *(ZE)*-bilirubin production under green light was less than or equal to that under blue light, and the effects of *(ZE)*-bilirubin on enterohepatic circulation were less than or equal to those under blue light. There were three patterns of the ratio of configurational isomers/*(ZZ)*-bilirubin: *(ZE)*-bilirubin/*(ZZ)*-bilirubin ratio > *(EZ)*-bilirubin/*(ZZ)*-bilirubin ratio, *(ZE)*-bilirubin/*(ZZ)*-bilirubin ratio < *(EZ)*-bilirubin/*(ZZ)*-bilirubin ratio, and *(ZE)*-bilirubin/*(ZZ)*-bilirubin ratio = *(EZ)*-bilirubin/*(ZZ)*-bilirubin ratio. These patterns in rat SA, rabbit SA, dog SA, bovine SA, pig SA, and human SA in this study were the same as those in a previous report using blue light^12^.

In animal SAs other than monkey serum and pig SA, cyclobilirubin production, which is important for the effects of phototherapy, was markedly lower than that in human SA. In pig SA, although the cyclobilirubin production rate was not significantly different from that in human SA, the ratio of *(EZ)*-bilirubin to *(ZZ)*-bilirubin was higher and ‘k’ was lower than in human SA. Therefore, pig SA was considered to have more *(EZ)*-bilirubin (Fig. 3), which is a precursor and a substrate of *(EZ)*-cyclobilirubin, resulting in cyclobilirubin production. This may aid in the development of green light sources to increase the efficiency of cyclobilirubin production. The cyclobilirubin production rate, ‘k’, and the ratio of *(EZ)*-bilirubin to *(ZZ)*-bilirubin in monkey serum were similar to those in human SA. The rhesus monkey may also be used as an animal model for the use of green light sources in phototherapy for human neonatal hyperbilirubinemia. Humans and rhesus monkeys are both primates that exhibit physiological jaundice in the neonatal period.

In this study of green LEDs, we found characteristic bilirubin photochemical changes in humans that may be helpful for the development of green light sources to increase the efficiency and safety of phototherapy.

This study has some limitations. First, regarding rhesus monkeys, as the photochemical reaction was carried out in serum, it is possible that the cyclobilirubin production rate was overestimated due to the presence of other substances dissolved in the serum compared with purified SA, but structural isomerization similar to that in human SA was also noted in rhesus monkey serum when green LEDs were used. Second, although *(EE)*-bilirubin is one of the configurational photoisomers, it was excluded from this study because it was present in a small amount and showed little effect in clinical phototherapy for neonatal hyperbilirubinemia^26^.

In conclusion, bilirubin photochemical kinetics with green LEDs in humans was characterized by a high cyclobilirubin production rate at a low precursor concentration. The photochemical kinetics of bilirubin in rhesus monkeys were similar to those in human SA.

## Methods

### Reagents and preparation

Bilirubin IXα (Tokyo Chemical Industry Co., Ltd., Tokyo, Japan) was used without further purification. SA from various species [human (essential free acid-free), rat (fraction V), rabbit (fraction V), dog (fraction V), pig (fraction V), sheep, bovine and chicken (fraction V)] was obtained from Sigma Aldrich Inc. (St Louis, USA). DMSO was purchased from Dojindo Laboratories (Kumamoto, Japan). Rhesus monkey serum (Lot 234) was purchased from Nippon Bio-test Laboratories Inc. (Saitama, Japan). The albumin concentration of the serum measured by the modified bromocresol purple method was 4.2 g/dL (650 μM).

Sample preparation and measurement were performed in a dark room. SA of each animal species, excluding the rhesus monkey, was dissolved in 0.1 M phosphate buffer (pH 7.4), and 2 g/dL solutions were prepared. Bilirubin was dissolved in 0.05 M sodium hydroxide and mixed with the SA solution of each animal species to prepare 10 mg/dL bilirubin-SA complex solutions (bilirubin/albumin molar ratio: 0.58). The molar ratio of bilirubin/rhesus monkey SA was 0.26. Bilirubin solution dissolved in DMSO (DMSO solution) at 10 mg/dL was used. Green LEDs (LF-111, Toitu, Osaka, Japan) were used as the light source. The LF-111 is a phototherapy device that emits 450–550-nm LED light centered at 500 nm. Bilirubin-SA complex solution (200 μL) was added to a test tube, which was kept standing on the light source, and the light irradiation time was set to 0, 1, 2, and 3 min. The irradiance was 24.3 μW/cm^2^/nm, as measured by a Minolta Air-Shield 451 Fluoro-Lite Meter. The bilirubin fraction was measured at each irradiation time point using HPLC ^27^. This process was repeated 3 times.

**Figure 4:**
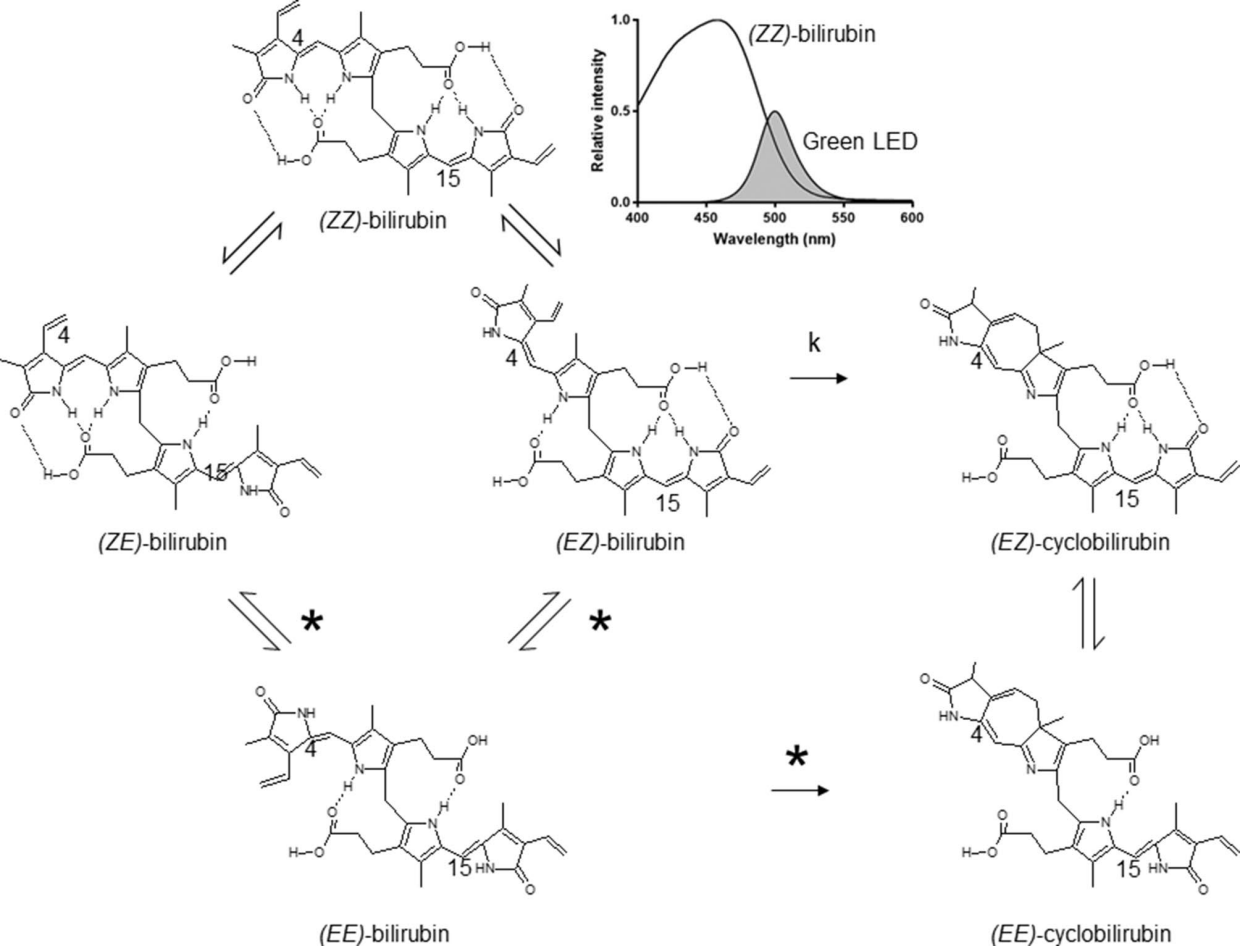
The structure and photochemical reactions of bilirubin and its photoisomers. The relative spectra represent the absorption spectrum of *(ZZ)*-bilirubin-human SA solution [phosphate buffer (pH 7.4)] and the irradiation spectrum of the green LEDs. The asterisk (*) indicates a theoretical pathway.

The structures of bilirubin and bilirubin photoisomers and their photochemical reaction are shown in Fig. 4. The absorption spectrum of *(ZZ)*-bilirubin-SA solution and the irradiation spectrum of the green LEDs are also shown. *(EE)*-bilirubin is one of the configurational photoisomers, but it was present only in a small amount^26^. The route involving *(EE)-*bilirubin may therefore be ignored. The absorption spectra of *(ZZ)*-bilirubin and its photoisomers vary greatly with albumin binding and dissolution. *(ZE)*-Bilirubin and *(EZ)*-cyclobilirubin in 0.1 M di-n-octylamine acetate in methanol (pH 7.6)^28^ and *(EZ)*-bilirubin in 0.15 M NaOH/1 mM Na_2_ EDTA/0.05 M HEPS (pH 7.4)^5^ were previously reported. When *(EZ)*-cyclobilirubin is produced from *(EZ)*-bilirubin, *(EZ)*-cyclobilirubin and *(EE)*-cyclobilirubin rapidly reach photoequilibrium^27^. Thus, the *(EZ)*-cyclobilirubin production rate was calculated as the cyclobilirubin production rate by dividing the sum of *(EZ)*-cyclobilirubin and *(EE)*-cyclobilirubin (as cyclobilirubin) by the irradiation time. When *(ZZ)*-bilirubin and *(EZ)*-bilirubin are at photoequilibrium, the relationship d [*(EZ)*-cyclobilirubin]/dt = ‘k’ [*(EZ)*-bilirubin] is established^12^. When the *(EZ)*-bilirubin/*(ZZ)*-bilirubin ratio is constant, *(EZ)*-bilirubin and *(ZZ)*-bilirubin are at photoequilibrium, and under this condition, *(EZ)*-cyclobilirubin and *(EE)*-cyclobilirubin are also at photoequilibrium^26,27^; therefore, [*(EZ)*-cyclobilirubin] = [cyclobilirubin] can be considered to hold. Accordingly, d[cyclobilirubin]/dt = ‘k’ [*(EZ)*-bilirubin], and `k’ was calculated from this formula (Fig. 4). As the production of *(ZE)*-bilirubin and *(EZ)*-bilirubin depends on *(ZZ)*-bilirubin because of their photoequilibrium properties, the *(ZE)*-bilirubin/*(ZZ)*-bilirubin and *(EZ)*-bilirubin/*(ZZ)*-bilirubin ratios as the production of *(ZE)*-bilirubin were used for comparison among animal species. The cyclobilirubin production rate, ‘k’, *(ZE)*-bilirubin/*(ZZ)*-bilirubin ratio, and *(EZ)*-bilirubin/*(ZZ)*-bilirubin ratio were calculated for each animal species.

### Bilirubin fraction measurement method

The bilirubin fraction was measured at each time point using HPLC as described previously^27^. A gradient elution reversed-phase HPLC system (Shimadzu LC-20AG, Shimadzu Co., Kyoto, Japan) with a UV/visible detector (PD-20AV detector, Shimadzu Co., Kyoto, Japan) was employed.

### Statistical analysis

Prism 7 for Windows version 7.02 (GraphPad, California, USA) was used for the statistical analysis. The cyclobilirubin production rate was analyzed using linear regression analysis, and the *(ZE)*-bilirubin/*(ZZ)*-bilirubin ratio, *(EZ)*-bilirubin/*(ZZ)*-bilirubin ratio, cyclobilirubin production rate, and ‘k’ of humans and animal species were compared using one-way analysis of variance followed by Dunnett's multiple comparisons test as a post hoc test, setting the significance level at 0.05.
